# Outcomes of retained gastrointestinal debris during upper endoscopy

**DOI:** 10.1055/a-2544-2468

**Published:** 2025-04-04

**Authors:** Jake Sheraj Jacob, Jeffrey Than, Christine Tang, Joseph Cano, Rehman Sheikh, Sharon Wolfson, Aaron P. Thrift, Uma Munnur, Robert J. Sealock

**Affiliations:** 1 Gastroenterology, Baylor College of Medicine, Houston, United States; 2 3270 Internal Medicine, Northwestern University, Evanston, United States; 3 Department of Medicine, Section of Gastroenterology and Hepatology, Baylor College of Medicine, Houston, United States; 4 Texas Digestive Disease Consultants, Flower Mound, United States; 5 3989 Department of Medicine, Baylor College of Medicine, Houston, United States; 6 3989 Department of Medicine, Section of Epidemiology and Population Sciences, Baylor College of Medicine, Houston, United States; 7 3989 Dan L Duncan Comprehensive Cancer Center, Baylor College of Medicine, Houston, United States; 8 3989 Department of Anesthesiology, Baylor College of Medicine, Houston, United States; 9 3989 Department of Medicine, Section of Gastroenterology and Hepatology, Baylor College of Medicine, Houston, United States

**Keywords:** Endoscopy Upper GI Tract, Motility / achalasia, Quality and logistical aspects, Performance and complications

## Abstract

**Background and study aims:**

Gastrointestinal debris retention (GIDR) during endoscopy can result in aborted procedures, intubation, and aspiration. GIDR has increased significance with uptake of glucagon-like peptide-1 receptor agonist (GLP-1RA) use. Outcome analysis is vital to risk-stratify patients with GIDR during endoscopy. Our study evaluated the effect of GIDR on endoscopic complications.

**Patients and methods:**

This was a retrospective review of patients who underwent endoscopy between May 2016 and December 2021 with documented GIDR. The study included 138 patients with GIDR and 275 controls. Propensity score matching between patients with GIDR and controls was performed in a 1:2 ratio based on age, sex, body mass index (BMI), and American Society of Anesthesiologists (ASA) status. T-tests and chi square tests were used to compare continuous and categorical variables.

**Results:**

The GIDR group was younger and had lower BMI, with no difference in sex, race, American Society of Anesthesiologists status, or use of monitored anesthesia care. GIDR was more frequently encountered when indications were abnormal imaging, pain, and pancreatico-biliary. Amount of GIDR was quantified as “large” in 37.7% of cases and size of debris was associated with rate of aborted procedures.

**Conclusions:**

Our study did not demonstrate a significant increase in post-procedure complications in patients with GIDR. Further, the GIDR group had higher rates of opiate use, which can guide stratification of retention risk.

## Introduction

Retained gastrointestinal debris is a common finding during upper endoscopy despite adequate pre-procedure fasting 1 . Recent increased use of glucose-like peptide-1 receptor agonist (GLP-1RA) for diabetes and obesity has heightened clinical significance of retained debris because GLP-1RAs have been shown to slow gastric emptying 2 . Although retained debris is a commonly encountered clinical scenario, there is a paucity of data regarding its clinical significance and impact on management and procedure complications. Studies to date have shown that retained gastrointestinal debris can result in incomplete examinations due to obstructed views, aborted procedures, and possibly intubation or delay in endoscopic therapy 1 . In addition to risk of respiratory events associated with sedation during endoscopy, retained debris further raises concern for pulmonary aspiration, a potentially life-threatening complication.

In patients at high risk for aspiration (higher American Society of Anesthesiologists [ASA] physical status, emergencies, gastric outlet obstruction, upper gastrointestinal bleed, esophageal stricture, impaired gastric emptying), potential for aspiration should be considered when planning for endoscopy, specifically when determining the level of sedation and whether to perform endotracheal intubation 3 . The literature describes risk of aspiration in patients undergoing esophagogastroduodenoscopy (EGD), potentially mediated by impairment of the gag reflex with local anesthetic, use of intravenous sedation, and/or splinting of the upper esophageal sphincter by the endoscope 4 5 . Colonoscopy may also increase risk of aspiration due to gas insufflation into the colon and/or manual abdominal pressure during the procedure 3 . Risk of aspiration also may be affected by the underlying health of the patient and the indication for the endoscopy. In Intensive Care Unit patients with upper gastrointestinal bleeding, Lipper et al. reports development of new lung infiltrates on chest x-ray after EGD, particularly those with fever and/or leukocytosis and oxygen desaturation below 90% during endoscopy 6 .

Although risk of aspiration in upper endoscopy has been well described, few studies have focused on patients with retained gastrointestinal debris during upper endoscopy or on procedures such as endoscopic retrograde cholangiopancreatography (ERCP) or endoscopic ultrasound (EUS). Our study aimed to evaluate whether gastrointestinal debris retention (GIDR) – defined as presence of debris as documented by the endoscopist – resulted in an increased number of aborted procedures, incomplete examinations, intubation, post-procedure imaging, Emergency Room (ER) visits, hospitalization, and aspiration pneumonia.

## Patients and methods

After Institutional Review Board approval was obtained, we conducted a single-center, retrospective analysis of 138 patients at a large county hospital in Houston, Texas who underwent upper endoscopy between May 2016 and December 2021 with documented GIDR. GIDR was defined as retained food seen in the esophagus, stomach, or duodenum during EGD, EUS, or ERCP. Endoscopists characterized GIDR into small, moderate, and large based on amount of lumen occupied by debris after insufflation (< 10%, 10%-33%, > 33%, respectively) with both forward-viewing and side-viewing endoscopes. Inpatients and outpatients who underwent EGD, EUS, and ERCP for a variety of emergent and non-emergent indications were included. Patients with mechanical obstruction and known gastroparesis were excluded from the study. These 138 patients were propensity-score matched in a 1:2 fashion with 275 patients using age, sex, body mass index (BMI), and ASA status, who had upper endoscopy performed during the same time period at the same facility, but without documented GIDR, as shown in Table 1. No further adjustments were made to the two cohorts after initial matching. Propensity score matching was done using R (version 4.3.3; R Core Team).

Patient data extracted from the electronic medical record included age at endoscopy, sex, race/ethnicity, BMI, hemoglobin A1c, presence of comorbid conditions including thyroid disorders and renal disease, history of opioid use, and history of psychiatric medication use. Charts were reviewed for chest imaging up to 2 weeks following endoscopy for any pulmonary complaint, and anesthesia records were reviewed for endotracheal intubation or peri-procedure complications. In addition, the medical record was reviewed for ER visits and/or hospitalizations up to 2 weeks following endoscopy.

Indications for endoscopy were divided into nine groups: abdominal pain, anemia, abnormal imaging, dysphagia, gastrointestinal bleed, gastroesophageal reflux disease (GERD), pancreatico-biliary disease, variceal screening, and other. The procedure was considered aborted if the endoscopist specifically documented such within the report or a complete examination was not documented. Our hospital’s Gastroenterology Anesthesia Department follows standard ASA guidelines for endoscopy.

### Statistical analysis

Student’s *t* -tests and chi square tests were used to compare continuous and categorical variables, respectively, between cases with GIDR and controls without GIDR. Central tendency and variability of the relevant variables were reported as mean and standard deviation, respectively. The Kruskal-Wallis test was used to compare means of more than two groups at once. Variables statistically significantly associated with GIDR ( *P* < 0.05) in univariable analysis were included in the multivariable logistic regression model. Statistical significance was considered *P* < 0.05. Analysis was performed using STATA 17.0.

## Results

### Baseline characteristics

Between May 2016 and December 2021, a total of 138 cases of GIDR during upper endoscopy were identified. Within the GIDR group, 107 procedures (78%) were performed under monitored anesthesia care (MAC) and 111 patients (80%) had an ASA of 3 or higher. Mean age of the GIDR group was 51.1 ± 15 and 76 patients (55%) were male. Distributions of age, sex, BMI, percentage of patients with ASA ≥ 3, percentage of cases performed under MAC, percentage of patients with hemoglobin A1c ≥ 7, procedure location (inpatient vs. outpatient) were not different between the GIDR group and controls. However, there was a higher proportion of patients with prior opiate use in the GIDR group when compared with controls. Patients in the GIDR group were less likely to have concurrent colonoscopy in the multivariable analysis. These data are summarized in Table 1 .

**Table:** **Table 1** Comparison of baseline characteristics between GIDR and control groups.

	GIDR (n = 138)	Control (n = 275)	Univariate analysis (P value)	Multivariate analysis (P value)
Age (mean ± standard deviation)	51.1 ± 15	53.8 ± 11	0.11	
Sex			0.92	
Male	76 (55%)	150 (55%)		
Female	62 (45%)	125 (45%)		
Race			0.42	
Hispanic	78 (57%)	133 (48%)		
Black	32 (23%)	70 (25%)		
White	22 (16%)	54 (20%)		
Asian	6 (4%)	18 (7%)		
Medical comorbidities				
Chronic kidney disease	19 (14%)	42 (15%)	0.73	
Opiate use	50 (36%)	23 (8%)	< 0.001*	< 0.001*
Thyroid disease	16 (12%)	47 (17%)	0.14	
Prior gastric surgery	3 (2%)	9 (3%)	0.53	
Hemoglobin A1c ≥ 7	22 (16%)	39 (14%)	0.63	
Psychiatric medication use	25 (18%)	66 (24%)	0.17	
Calcium channel blocker use	19 (14%)	48 (17%)	0.15	
BMI (mean ± standard deviation)	27.1 ± 6.4	27.9 ± 8	0.25	
ASA ≥ 3	111 (80%)	209 (76%)	0.31	
MAC	107 (78%)	217 (79%)	0.75	
Upper endoscopy + colonoscopy	10 (7%)	54 (20%)	< 0.001*	0.02*
Location			0.12	
Inpatient	73 (53%)	123 (45%)		
Outpatient	65 (47%)	152 (55%)		
Indication for upper endoscopy				
**Abnormal Imaging**	12 (9%)	9 (3%)	0.02*	0.03*
Anemia	7 (5%)	31 (11%)	0.04*	0.99
Abdominal pain	27 (20%)	19 (7%)	< 0.001*	0.47
Dysphagia	29 (21%)	24 (9%)	< 0.001*	0.01*
Gastrointestinal bleeding	29 (21%)	68 (25%)	0.4	
GERD	4 (3%)	24 (9%)	0.03*	0.19
Pancreatico-biliary	19 (14%)	3 (1%)	< 0.001*	0.004*
**Variceal screening**	11 (8%)	71 (26%)	< 0.001*	0.143
**Other**	18 (13%)	26 (9%)	0.26	
ASA, American Society of Anesthesiologists; GIDR, gastrointestinal debris retention; GERD, gastroesophageal reflux disease; MAC, monitored anesthesia care. *P < 0.05. Values reported as number of patients (%) unless specified.				

### Indication

The most commons indication for endoscopy in the GIDR (21%) group were gastrointestinal bleeding and dysphagia, and in the control group variceal screening (26%) ( Table 1 ). A greater percentage of GIDR patients underwent endoscopy for abnormal imaging, abdominal pain, dysphagia, and pancreatico-biliary indications. The control group had higher proportions of patients requiring endoscopy for anemia, GERD, and variceal screening ( *P* < 0.001).

### Gastrointestinal debris retention

Endoscopic documentation of quantity of retained debris included 52 with a large amount, 15 with a moderate amount, 16 with a small amount, and 55 cases in which the quantity was not documented ( Table 2 ). The most common location of GIDR was in the stomach, although five patients had debris isolated in the duodenum or in both the duodenum and stomach and 23 had debris isolated in the esophagus or in both the stomach and the esophagus.

**Table:** **Table 2** Comparison of amount of debris, aborted procedures, and post-procedure intubation between GIDR and control groups.

	GIDR (n = 138)	Control (n = 275)	P value
GIDR amount			
Large	52 (38%)	–	–
Moderate	15 (11%)	–	–
Small	16 (12%)	–	–
Unknown	55 (40%)	–	–
**Aborted procedure**	**45 (33%)**	**2 (1%)**	< 0.001*
**Post-procedure intubation**	**5 (3.6%)**	**0 (0%)**	0.001*
*P < 0.05. Values reported as number of patients (%). GIDR, gastrointestinal debris retention.			

### Aborted procedures

A higher percentage of procedures were aborted among patients with GIDR compared with controls ( *P* < 0.001, Table 2 ). In the control group, procedures of only two patients (1.6%) were aborted, both due to apnea and bradycardia. Neither patient had chest imaging in the 2 weeks following their endoscopy and both were treated as inpatients.

In contrast, 45 procedures (33%) in patients with GIDR were aborted. One procedure was aborted for a small amount of debris (6.3% of cases with small amount of GIDR), three for a moderate amount (20%), 24 for a large amount (46%), and 17 for an undocumented amount (31%) (H = 10.14, 2 degrees of freedom, *P* < 0.01). Of these cases, 10 (22%) had follow-up chest imaging within 2 weeks of endoscopy.

### Intubation

More patients with GIDR (3.6%) required post-sedation intubation compared with controls (0%, *P* < 0.001, Table 2 ). Among controls, two patients were intubated prior to the procedure for hematemesis. Of the GIDR patients, 18 patients required intubation, of whom only five were intubated after administration of procedure sedation. Inidcations for intubation during the procedure were dysphagia (achalasia), food impaction, hematemesis, and other (duodenal cancer).

### Chest imaging within 2 weeks

In patients who had imaging performed, we observed similar distributions of those who received chest imaging for complaints related to aspiration such as cough, fever, and dyspnea between the GIDR group and controls ( *P* = 0.38). A higher percentage of patients in the GIDR group (n = 30, 22%) had chest imaging performed 2 weeks after upper endoscopy compared with controls (n = 40, 15%), although the test for difference did not reach statistical significance ( *P* = 0.07, Table 3). In addition, there was no difference between the percentage of GIDR patients with radiological findings consistent with aspiration pneumonia (7.2%) compared to controls (5.1%; *P* = 0.16).

### Emergency room visits and hospitalizations

There was no difference in the number of patients who had ER visits ( *P* = 0.15) or hospitalizations ( *P* = 0.22) between the two groups ( Table 3 ). In addition, many of the ER visits were unrelated to endoscopy.

**Table:** **Table 3** Comparison of chest imaging and return to care within 2 weeks after endoscopy between GIDR and control groups.

	GIDR (n = 138)	Control (n = 275)	P value
Chest imaging within 2 weeks			
Total patients who received imaging	30 (22%)	40 (15%)	0.07
Imaging for symptoms of possible aspiration	10 (7.2%)	14 (5.1%)	0.38
Imaging with possible aspiration	10 (7.2%)	11 (4.0%)	0.16
Return to care within 2 weeks			
ER visits	14 (10%)	17 (6.2%)	0.15
Hospitalization	10 (7.2%)	12 (4.4%)	0.22
*P < 0.05. Values reported as number of patients (%). ER, Emergency Room; GIDR, gastrointestinal debris retention.			

### Dysphagia subgroup

Analysis was repeated for patients within the dysphagia subgroup. The percentage of patients who had small (9.4% vs. 3.1%) or unknown (28% vs. 11%) amounts of GIDR was higher among those undergoing endoscopy for dysphagia compared with other indications. Over one-quarter of patients with dysphagia (26%) received chest imaging within 2 weeks compared with 16% of other patients. No other outcomes were differentially distributed within the dysphagia subgroup ( Table 4 ).

**Table:** **Table 4** GIDR and outcome analysis for dysphagia subgroup.

	Dysphagia (n = 53)	No dysphagia (n = 360)	P value
GIDR amount			
Large	7 (13%)	45 (13%)	0.885
Moderate	2 (3.8%)	13 (3.6%)	0.953
Small	5 (9.4%)	11 (3.1%)	0.025*
Unknown	15 (28%)	40 (11%)	0.001*
Aborted procedure	7 (13%)	40 (11%)	0.654
Intubated	5 (9.4%)	15 (4.2%)	0.095
Chest imaging within 2 weeks			
Total patients who received imaging	14 (26%)	56 (16%)	0.049*
Imaging for symptoms of possible aspiration	5 (9.4%)	19 (5.3%)	0.227
Imaging with possible aspiration	5 (9.4%)	16 (4.4%)	0.123
Return to care within 2 weeks			
ER visits	4 (7.6%)	27 (7.5%)	0.990
Hospitalization	3 (5.7%)	19 (5.3%)	0.908
*P < 0.05 ER, Emergency Room; GIDR, gastrointestinal debris retention.			

## Discussion

This study provides evidence of the association between GIDR and an increased number of aborted procedures and intubations. GLP-1 RA use has been shown to increase rates of GIDR in retrospective analysis 7 , prompting societies to swiftly issue guidance on their management in the peri-endoscopic setting. Currently, there is conflicting guidance from the ASA, which suggests holding GLP-1RA 1 week prior to endoscopy in those with weekly dosing, and the American Gastroenterology Association, which counsels that most patients can be continued on their GLP-1RA prior to endoscopy 8 9 . These discrepancies highlight the need for data regarding outcomes in patients with GIDR.

In our analysis, there was a significant association between amount of debris and percentage of aborted GIDR cases, with only 6.3% of small debris cases requiring cancellation compared with 46% of large debris cases (H = 10.14, 2 degrees of freedom, *P* < 0.01). All the procedures that required intubation after procedure sedation were those with GIDR. The association between amount of debris and rate of aborted procedures may provide endoscopists with additional data to stratify risk.

Despite the association between GIDR and peri-procedure complications, the study found no significant difference between the two groups in overall rates of post-procedure imaging or incidence of post-procedure imaging consistent with aspiration. In patients with same-session EGD and colonoscopy, these rates also are no different between the two groups, although the sample size was small. These findings may be influenced by the higher rate of aborted procedures in the GIDR group. On the other hand, subgroup analysis of patients with dysphagia showed higher rates of a small amount of GIDR and need for chest imaging in the 2-week post-procedure period.

The GIDR group had a greater proportion of patients with opiate use when compared with controls ( *P* < 0.001). However, use of other medications that delay gastric emptying, such as calcium channel blockers and psychiatric medications, was similar between the two groups. Interestingly, a retrospective study identifying predictors of endoscopic food retention found that prokinetic agents such as metoclopramide and erythromycin similarly had no effect on odds of gastric retention 10 . These findings may impact clinical decision-making and stratification of retention risk in patients prior to endoscopy.

The study investigated patients undergoing upper endoscopy for both emergent and non-emergent indications. The most common indication for endoscopy in the GIDR group was gastrointestinal bleeding and dysphagia. The rate of debris retention in gastrointestinal bleeding may be related to a shorter time-to-endoscopy, given the relative urgency of the procedure when compared with other indications. Given the number of patients with dysphagia and GIDR, as well as increased rates of imaging in the post-procedure period in patients with dysphagia, our findings suggest that patients with complaints of dysphagia may benefit from more prolonged NPO status or alternative diet, such as a liquid diet, prior to the procedure.

A limitation of this study is the estimation required in quantifying the amount of retained debris and in the risk:benefit ratio for continuing endoscopy during these cases. In aborted procedures, patient safety precluded completing the exam and documenting whether debris was present distal to the extent of the exam. Procedure abortion was evaluated through endoscopic reports and was not validated in person. The retrospective nature of this study also limits comparison of baseline characteristics such as hemoglobin A1c, given the availability of data in patient charts. Additional prospective studies may be warranted to further elucidate the role of GIDR in procedure complications. The time period examined was before uptake of GLP-RA use, limiting our analysis of the effects of these medications on procedure outcomes.

## Conclusions

In our single-center retrospective study, we investigated outcomes of patients found to have retained gastrointestinal debris during upper endoscopy. Our data support increased rates of intubation and aborted procedures in patients with GIDR compared with patients without GIDR on endoscopy. However, patients with GIDR did not have significantly higher rates of post-procedure complications–namely imaging rates, radiographic evidence of aspiration, hospitalizations, and ER visits – within 2 weeks of endoscopy. Interestingly, rates of aborted procedures increased with the volume of retained debris. These findings may help guide decision-making in patients found to have retained debris on upper endoscopy and help liberalize management of GLP-1 RA in the periprocedure period.
